# FlavorGraph: a large-scale food-chemical graph for generating food representations and recommending food pairings

**DOI:** 10.1038/s41598-020-79422-8

**Published:** 2021-01-13

**Authors:** Donghyeon Park, Keonwoo Kim, Seoyoon Kim, Michael Spranger, Jaewoo Kang

**Affiliations:** 1 Department of Computer Science and Engineering, Korea University, Seoul, 02841 South Korea; 2 SONY AI, SONY Corporation, Tokyo, 108-0075 Japan

**Keywords:** Applied mathematics, Computer science, Scientific data, Statistics, Biochemistry, Mathematics and computing, Health care, Quality of life

## Abstract

Food pairing has not yet been fully pioneered, despite our everyday experience with food and the large amount of food data available on the web. The complementary food pairings discovered thus far created by the intuition of talented chefs, not by scientific knowledge or statistical learning. We introduce *FlavorGraph* which is a large-scale food graph by relations extracted from million food recipes and information of 1,561 flavor molecules from food databases. We analyze the chemical and statistical relations of *FlavorGraph* and apply our graph embedding method to better represent foods in dense vectors. Our graph embedding method is a modification of metapath2vec with an additional chemical property learning layer and quantitatively outperforms other baseline methods in food clustering. Food pairing suggestions made based on the food representations of *FlavorGraph* help achieve better results than previous works, and the suggestions can also be used to predict relations between compounds and foods. Our research offers a new perspective on not only food pairing techniques but also food science in general.

## Introduction

Food pairing has been one of the key topics in food science and is currently an essential task in culinary practice. Despite the efforts of chefs, gourmets, and researchers to discover new food pairings, there are still pairings that have yet to be revealed in the culinary world. To master food pairings, one must have a clear understanding of food itself. However, understanding food is a difficult task as it has many descriptive features such as flavor, color, texture, and so on. Many researchers sought to tackle this problem by making the most of their data such as millions of food recipes. Finding the best representations of foods will be helpful in discovering better food pairings. Here, we aim to answer the following two questions: How can we utilize the available data and meaningful descriptive features to obtain better food representations? How can we apply improved food representations to food pairing? To address these questions, we introduce *FlavorGraph* which is a large-scale network of food ingredients and chemical compounds. A graph embedding method called metapath2vec with an additional chemical learning layer is applied to construct embedded representations of food, which are used in our food pairing task. The results show that the food representations are applicable to food pairing and can help in suggesting novel pairings.

Previous works have proposed various chemical-based approaches to help improve food pairing. Ahn et al.^1,2^ introduced a flavor network where the edges in the network are created based on the number of flavor compounds shared by culinary ingredients. FlavorDB^3^ combines existing food repositories to provide a larger database with a user-interactive page. Food-bridging^4^ improves the flavor network of^1^ by adding additional bridges between two ingredients through a chain of pairwise affinities even though the chemical compound similarity of the two ingredients is low. However, one critical limitation of the chemical-based approaches is that the number of foods and flavor molecules investigated in previous studies is very limited. Performing food-chemistry experiments (e.g., Gas Chromatography) is very expensive. In addition, it is difficult to accurately represent the chemical compounds of foods in a form that can be stored digitally because there are various features (e.g., flavor, color, texture, smell) and different varieties of the same food. Incorporating flavor compound information is indeed fundamental in food pairing. However, the lack of available chemical information of food makes it difficult for chemical-based approaches to construct accurate food representations in food pairing tasks.

Several recipe-based approaches which involve using recipe collections have also been previously proposed for food pairing tasks. Teng et al.^5^ proposed a recipe recommendation approach that uses ingredient networks to determine whether a food ingredient is essential in a recipe. This approach uses two different recipe networks to find which ingredients go well together or can be used as substitutes for better recipes. There have been also studies on the analysis of food preference and food pairing according to regional characteristics each in China^6^ and India^7^. Researches on food preferences done by Wagner et al.^8^, Abbar et al.^9^ focused on studying personal food preferences using online user data. On top of that, Zhang et al.^10^ proposed the restaurant recommendation to guide dining preferences based on the user’s food history. Other approaches^11,12^ that combine case-based reasoning and deep learning for automatic recipe generation have been introduced. KitcheNette^13^ uses deep Siamese neural networks trained on a large recipe dataset to predict food pairing scores. The hidden representations from the shared embedding layer of KitcheNette are used for predicting the co-occurrence of food ingredients in recipes and contribute to discovering novel food pairings by referencing similar food representations. However, as these approaches are solely based on statistical co-occurrence among many recipes, chemical compound information is not taken account in constructing food representations and recommending food pairing.

Many studies have been made to construct different types of representations using data-driven approaches and semantic concepts employed in other research fields. Semantic concepts are coherent ideas that come from words in language, people nodes in social networks, entities in databases, and so on. Word2vec^14,15^ is a neural network that learns distributed representations of word vectors trained on textual data (sentences). In food research, Im2recipe^16^ utilized word2vec to create food representations based on a large corpus of recipes. These food representations were extensively used for inferring food images^16^ and generating novel food recipes^17^. Reciptor^18^ most recently proposed a set transformer-based model to obtain recipe embeddings and uses a knowledge graph (KG)^19^ derived triplet sampling approach to optimize the learned embeddings.

In graph-based embedding approches, node2vec^20^ has been employed for building node representations from network data. Node2vec has also been used in network analysis and graph mining tasks. Node2vec generates random walks according to the relations of its network where the walks are analogous to sentences in word2vec. The nodes of the network are trained on each walk where the neighboring nodes serve as contextual information. Metapath2vec^21^ which generates heterogeneous node representations using large-scale networks has been recently introduced. The random walks called metapaths generated by metapath2vec^21^ are used for constructing similar representations of heterogeneous nodes based on commonly linked nodes. These data-driven and graph-based approaches that embed conceptual representations with rich domain-specific information may improve food pairing recommendations as the approaches can be used to construct food representations based on the relations between different foods and chemical compounds.

**Figure 1:**
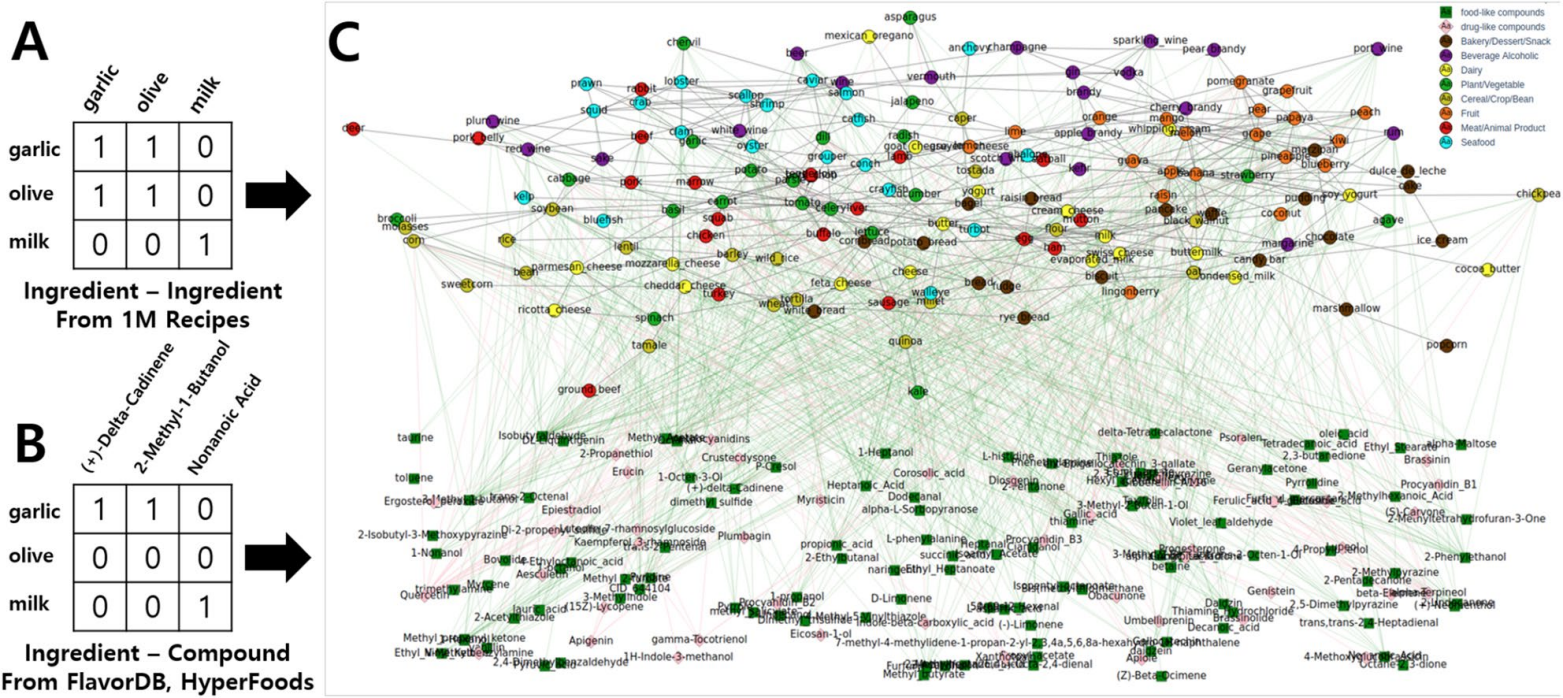
FlavorGraph. (**A**) Ingredient-ingredient relation. The relations between ingredients are shown; two ingredients are a “good pair” if they are used together in a large number of food recipes. The relations were obtained from Recipe1M^22^. (**B**) Ingredient-compound relation. The relations between ingredients and chemical compounds are shown. These relations were obtained from FlavorDB^3^ and HyperFoods^23^. (**C**) A partial view of *FlavorGraph*. Only *160 out of 6653* ingredients, *154 out of 1646* compounds, and their relations are shown in Fig. 1 for better illustration. Note that the whole graph was used for model training.

Using the approaches of previous studies, we built *FlavorGraph* (Fig. 1) which is a large-scale graph network of food and chemical compound nodes. *FlavorGraph* (Fig. 1) contains 6653 food ingredient nodes and 1561 food-related chemical compound nodes, 84 drug-like chemical compound nodes, and two relations among them. First, the relations between ingredients and ingredients (111,355 edges) are based on their probability of being used together (NPMI, Normalized Point-wise Mutual Information) in 1 million recipes (Fig. 1A). The relations between ingredients and chemical compounds (35,440 edges) were obtained from food-related academic resources that specify such food-compound relationships (Fig. 1B).

Then we adopted the graph node embedding method of metapath2vec^21^ with an additional chemical structure learning layer to make conceptual representations of food. A metapath is a predefined sequence of node types which are connected as edges according to the graph. Sub-graphs that satisfy the metapath condition were randomly selected from *FlavorGraph*. The embedded node vectors of *FlavorGraph*, which are food representations, were trained in a skip-gram fashion based on their sub-graph connectivity. Food ingredient nodes are further divided into two types: ‘chemical-hub ingredient nodes’ and ‘non-hub ingredient nodes’. Among the ingredient nodes, we refer to the nodes that have a relation with the chemical compound nodes as ‘chemical-hub ingredient nodes’. We found that only 416 food ingredient nodes (chemical-hub ingredient nodes) of the 6653 food ingredient nodes have chemical information, but the remaining 6237 nodes (non-hub ingredient nodes) do not. A small portion of food ingredients having such chemical information can lead to several model optimization issues. To overcome this issue, we employ two methods in this study. First, we created food-specific metapaths^21^ where all three types of nodes are involved. Our intuition is to enforce the embedding model to pass the information from chemical compound nodes to non-hub ingredient nodes via chemical-hub ingredients nodes in a single metapath. Second, upon food pairing relations regarding the chemical information of food ingredients (35,440 edges), we also utilized the statistical co-occurrence of two food ingredients (111,355 edges). Therefore, we designed a model that considers not only the chemical aspects of food pairing but also the statistical aspects as well.

To further elaborate on food-specified metapaths, we created multiple to ensure that chemical information from compound nodes is passed to non-hub ingredient nodes via chemical-hub ingredient nodes. The chemical-hub ingredients nodes act as important intermediary nodes between two disjoint heterogeneous nodes (e.g., flavor compounds to chemical-hub ingredients to non-hub ingredients) and learn as much chemical as possible. Likewise, the created metapaths help food representation vectors simultaneously train on complex relations such as food–food (e.g., red_wine&steak) and food-chemistry (e.g., red_wine&ractone) relations. The final number of metapaths was 1,114,285 and the average length of them was 46.4. We then trained these metapaths in skip-gram fashion to obtain meaningful food representations. The food representations were further used in many downstream tasks including food pairing. The results of food representations are shown in Fig. 3.

We conducted quantitative experiments and a qualitative analysis to evaluate whether the food representations constructed using the metapath-based graph embedding method of *FlavorGraph* provide meaningful information and help improve performance in food pairing tasks. Compared with other food embedding methods, our metapath-based graph embedding method with a chemical structure learning layer achieved the highest score in a node clustering task of correctly categorizing each food. We also performed a similarity search based on the cosine similarity ranking of the food representation vectors. We assumed that our method would yield food pairings that are chemically similar and/or used in the same recipe context. In practice, for a given query, our embedding method provides ranked results of recommended parings of ingredients that are chemically similar and used in the same recipe. The trained food and chemical representations can be further used with simple mathematical operations to suggest food pairings. In addition, the ranked results of recommended pairings can be used to provide a list of foods related to a particular food molecule or drug compound. In summary, our food representations are meaningful as they helped to improve performance in food clustering and food pairing recommendation.

The major contributions of this work are summarized as follows.

- We introduce *FlavorGraph* which is a large-scale network graph built from recipe and chemical relations (147,179 edges) of food ingredients (6653 nodes) and chemical compounds (1646 nodes).
- We propose a food-specialized graph embedding method that constructs meaningful food representations for *FlavorGraph*, and demonstrate that our method outperforms other methods in a node clustering task which involves categorizing features of food.
- We demonstrate the effectiveness of our food representations in the tasks of food pairing recommendation and food-compound relation prediction.

## Results and discussion

### Preliminary analysis on food pairings with chemical compound information and recipe co-occurrence

We performed preliminary analysis on the large amount of recipe data from Recipe1M^22^ and chemical information in foods from FlavorDB^3^ as both datasets are involved in this study. We first examined the relevancy between chemical compound information in foods and their category labels. Next, we looked into the correlation between pairwise ingredient co-occurrence probability in 1 million recipes and pairwise chemical similarity based on the number of overlapping chemical compounds in two ingredients. We denote the number of overlapping chemical compounds as chemical overlaps.

**Figure 2:**
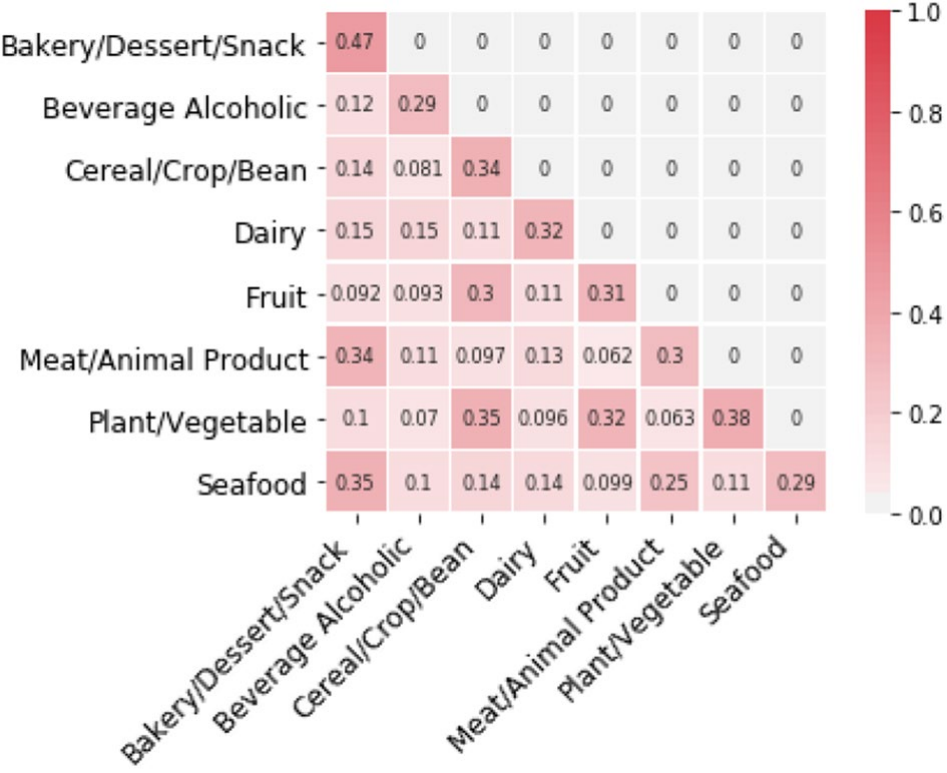
Chemical structure similarity between two categories. The scores in each box refer to the mean of all the Jaccard similarity scores of two ingredient chemical vectors within their categories. The ingredient chemical vectors are in one-hot vector fashion, which represents the presence of chemical compound in each ingredient.

*Relation between chemical information and category labels of food ingredients* Referring to Fig. 2, we found the food ingredients in same categories tend to have similar chemical structures. The scores in each box in Fig. 2 refer to the mean value of all Jaccard similarity scores of two ingredient chemical vectors within their categories. Each position in the ingredient chemical vectors represents the presence of a chemical compound (0 or 1). We observed that the similarity scores within same categories are largely high except for few corner cases. The mean value of similarity scores between same categories are 0.324 while different categories yielded the mean value of 0.167.

*Relation between recipe co-occurrence and chemical similarity of two food ingredients* In Table 1, we examined the following two hypotheses regarding our *FlavorGraph* data: (1) Whether the co-occurrence probability of two ingredients in a large corpus indicate good pairings. (2) Whether the high number of overlapping chemical compounds indicate good pairings.

**Table 1:** Relation between recipe co-occurrence based pairing ranking and chemical overlap based ranking.

Ingredient 1	Ingredient 2	Co-occur Prob.	Chemical overlap
**Recipe co-occur. ranking**			
bamboo_shoot	water_chestnut	0.532	94
Parsnip	Turnip	0.524	101
Oregano	Basil	0.515	159
Raspberry	Blackberry	0.503	116
Nutmeg	Cinnamon	0.472	139
Miso	Sake	0.507	4
Anchovy	Caper	0.415	2
Saffron	basmati_rice	0.394	6
ham	swiss_cheese	0.349	2
tortilla_chip	Avocado	0.317	5
**Chemical overlap ranking**			
Apple	Strawberry	0.021	189
Bean	green_bean	0.117	178
Banana	Apple	0.090	173
Pineapple	Apple	0.081	169
Apricot	Apple	0.069	169
Cocoa	Apple	− 0.178	167
Orange	mandarin_orange	0.054	166
Papaya	Apple	− 0.049	165
Ginger	Pepper	− 0.026	164
Lemon	mandarin_orange	− 0.066	164

Co-occurrence probability is normalized point-wise mutual information (NPMI) between pairs of ingredients. NPMI ranges from − 1 to 1, with − 1 (never occurred together), 0 (independent of each other), and 1 (co-occurred perfectly).

For the first hypothesis, we actively selected 10 ingredient pairs among the top-ranked pairs with high recipe co-occurrence probability (NPMI). Some pairs have large chemical overlaps (e.g., bamboo_shoot&water_chestnut, oregano&basil, raspberry&blackberry and so on). These two ingredients are well-known *“Congruent Pairings”*^24^, where they share many chemical compounds and have high co-occurrence in many recipes. On the other hand, some frequently co-occurring pairs have small chemical overlaps. For example, miso&sake, anchovy&caper and saffron&basmati_rice are ingredients pairs that are chemically dissimilar as they have few shared compounds, but are well-known good pairings. For these cases, these types of pairings are called as *“Complementary Pairings”*^24^. For the second hypothesis, we ranked the 10 samples of ingredient pairings by their chemical overlaps. The top ranked pairings are mostly similar types of foods (e.g., fruit–fruit and bean–bean), but with quite low co-occurrence probabilities (− 0.178 0.117). We found that high chemical overlaps lead to chemically related foods in similar categories but do not necessarily mean good pairings.

To wrap up our preliminary analysis, we confirmed again that it is important to incorporate both recipe co-occurrence information and chemical information for building *FlavorGraph*. For improved food representations, our approach for building the metapaths focuses on propagating both types of information to the embedded nodes in our constructed *FlavorGraph*.

### Representing food ingredients and chemical compounds in a vector space

**Figure 3:**
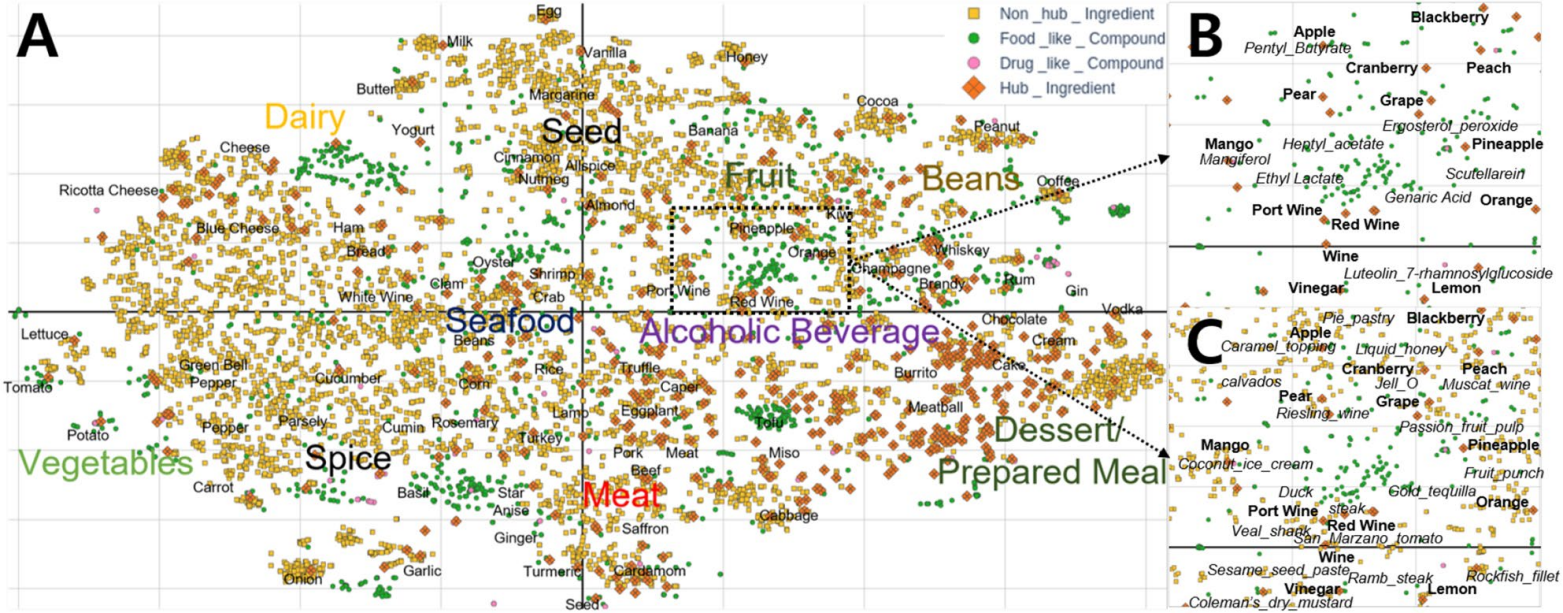
FlavorGraph node representation results. (**A**) 2D t-SNE projection of whole food ingredient nodes (6653) and chemical compound nodes (flavor compounds: 1561, drug compounds: 84) from *FlavorGraph*. (**B**) <chemical hub ingredient-compound relation>. This figure shows the chemical compounds shared by wines and citrus fruits (orange, pineapple, grape, cranberry) and how they affect their overall taste. (**C**) <chemical hub ingredient-non hub ingredient>. This figure shows which common foods go well with certain wines and citrus fruits.

Figure 3A shows the results of applying the graph embedding to *FlavorGraph*. Using metapath2vec^21^, we generated user-specified metapaths to learn the chemical relations between foods and chemical compounds, and how foods have been used together in recipes. A metapath is a path where heterogeneous nodes (food, compound, and drug) are connected to each other based on their relations in *FlavorGraph*. The <Food-Compound> relations are from two distinguished food databases^3,23^. The <Food-Food> relations are identified by the statistical co-occurrence of food ingredients in one million recipes^4^. Further details on generating and learning metapaths are provided in the Methods section.

Figure 3A shows the 2D t-SNE projection of heterogeneous node representations (food ingredient nodes [chemical hub or non hub nodes], flavor compound nodes, and drug compound nodes). Food nodes are further divided into chemical hub ingredient nodes (indicated by orange diamonds, 6% of total ingredients) that have relations to chemical compounds, and the remaining ingredient nodes (indicated by yellow squares, 94% of total ingredients). Certain clusters of nodes are formed around these chemical hub ingredient nodes according to the nodes food category (e.g. fruit, dairy and so on). In each cluster, the chemical hub ingredient nodes are located closely to the chemical compound nodes (indicated by green and pink dots) which are included in the chemical hub ingredient nodes. Also, there are many food ingredient nodes with chemical information (indicated by yellow squares) near the chemical hub ingredient nodes, which indicates that the food ingredients are likely to be used together in recipes. Figure 3B shows that the wine and citrus fruit nodes are located closely to each other and that the wines and fruits share flavor compounds that are actually part of their composition (e.g., red_wine&genaric_acid and red_wine&ethyl_lactate). Figure 3C shows what kinds of food would go well with wines and citrus fruits (e.g., red_wine&steak and red_wine&marzano_tomato). The results show that if two food ingredient nodes are close to each other in the vector space, they may be a good pair as they share similar chemical structures and have a high possibility of being used in the same recipe.

### Node clustering by food category

To illustrate how well our food representations align with their categorical distribution, we conducted a node clustering task. We measured the clustering accuracy with Normalized Mutual Information (NMI) as done in the work by Dong^21^. Originally, the Mutual Information (MI) is a measure of the similarity between two labels of the same data. Here, the two labels are predicted clustering labels and actual clustering labels. The Normalized Mutual Information (NMI) is a normalization of the Mutual Information (MI) score to scale the results between 0 (no mutual information) and 1 (perfect correlation). The results of the node clustering task are shown in Table 2.

**Table 2:** Node clustering results.

Model	Random	FlavorDB	Im2Recipe	node2vec/DeepWalk	metapath2vec	metapath2vec+CSP
Method	–	Number of shared flavor molecules	Text embedding	Heterogeneous random walk paths	Probabilistic meta-paths	Probabilistic meta-paths + Chemical learning
Dimension	–	1645 (binary)	300 (dense)	300 (dense)	300 (dense)	300 (dense)
Node Clustering (NMI)	0.111	0.272	0.079	0.286	0.286	*0.309

The scores indicate for normalized mutual information (NMI). In the case of FlavorDB, since there is no continuous and dense vector, 1645 binary vectors representing the presence or absence of each chemical compound were used.

*We found significant differences among all the results where ${p < .05}$ and the number of sampled results ($n = 1000$).

For the clustering categories, we then collected the cuisine categories of 416 chemical hub ingredients from FlavorDB^3^. Originally, the total number of categories in FlavorDB is 34. Instead of using all the information, we chose to merge them into the following representative nine food categories: Bakery/Dessert/Snack, Beverage Alcoholic, Cereal/Crop/Bean, Dairy, Fruit, Meat/Animal Product, Plant/Vegetable, Seafood, and Others. The statistics of each cuisine category is shown in Table 3. Note that we only used 416 chemical hub ingredients for fair comparison since they were commonly used in all other models.

**Table 3:** Cuisine category statistics of over FlavorGraph.

Category	# of ingredients
Bakery/dessert/snack	44 (10.3%)
Beveraage alcoholic	24 (5.8%)
Creal/crop/bean	38 (15.4%)
Dairy	6 (7.2%)
Fruit	15 (13.5%)
Meat/animal product	19 (4.6%)
Plant/vegetable	128 (30.8%)
Seafood	31 (7.45%)
Others	21 (5.05%)
Total	416

We collected the 416 chemical hub node ingredients, which co-exist in FlavorDB and other baselines models where they contain chemical compound information for category clustering.

We compared the performance of the our modified version of metapath2vec on *FlavorGraph* with that of five different food vector embedding methods in clustering food ingredient nodes. To create food vectors for FlavorDB, we used the occurrence of chemical compounds in each food to represent a position of a binary vector (1645-D). For food vectors of Im2recipe, we utilized the word embedding^14^ results of one million recipes on which a simple skip-gram model was trained. For the food vectors of node2vec and metapath2vec, and the *FlavorGraph*, we used different graph node embedding methods for each. Node2vec uses a simple random walk and metapath2vec uses probabilistic metapath walks. The graph embedding applied to *FlavorGraph* also uses metapath2vec but has an additional chemical embedding layer for better representing ingredient nodes. The flavor representations of *FlavorGraph* showed the best result (NMI score of 0.309) in node clustering. The hyperparameter setting of this experiments is further discussed in M1 of Supplementary Information.

**Figure 4:**
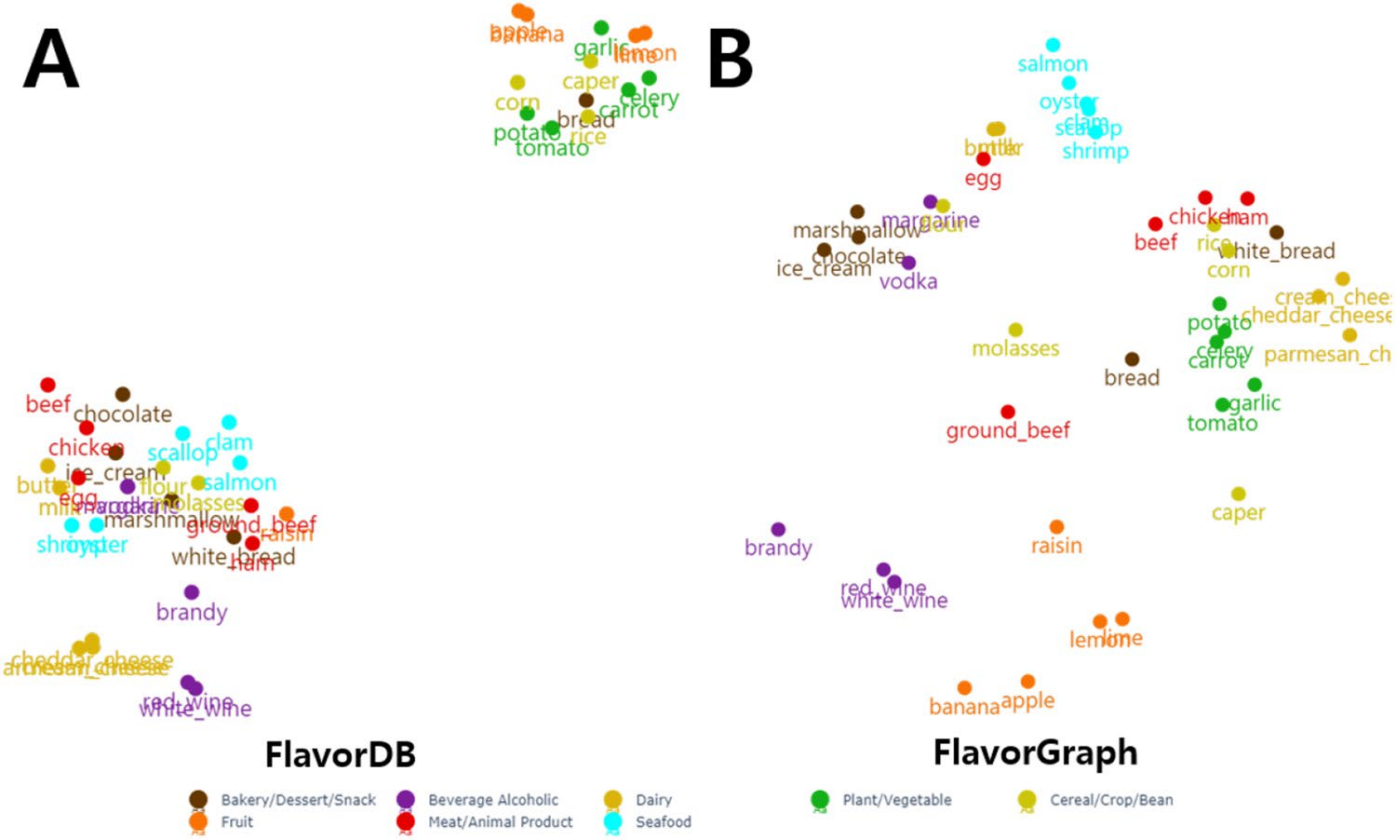
2D t-SNE projection of the 300-D embeddings of 40 food ingredients, five from each of the eight food categories. (**A**) FlavorDB (**B**) *FlavorGraph*.

Figure 4 shows the 2D t-SNE projection of FlavorDB node representations (A) and that of *FlavorGraph* node representations (B). As shown in Fig. 4A, clustering result seems to be effective, but similar categories slightly overlap. Moreover, the chemical properties of foods in the vegetable and fruit categories are different from those of foods in the other categories. On the other hand, as the clustering result in Fig. 4B shows, all food nodes of each food category are distributed evenly according to their category.

### Case Study 1: using the flavor representations of *FlavorGraph* for food pairing

In Table 4, as done in KitcheNette^13^, we selected four popular food ingredients (tomato, onion, pepper, cinnamon) and performed a similarity search based on the query ingredient. The similarity search results of the flavor representations of *FlavorGraph* can be used to provide two different food pairing recommendations. The chemical recommendation of food pairings (left table in Table 4) demonstrates that *FlavorGraph* representations can be used to recommend food pairings if foods are chemically complementary. FlavorDB’s ranking of four foods (e.g., FlavorDB ranking of tomato: https://cosylab.iiitd.edu.in/flavordb/entity_details?id=364) in ascending order is based on the number of chemical molecules shared by a pair of food ingredients. Most food ingredients in both ranking results of *FlavorGraph* and FlavorDB share many chemical molecules with the four given food ingredients. However, some food ingredients in the *FlavorGraph* ranking results share extremely few chemical molecules (e.g., jalapeno (0), and red_bell_pepper (0)). The graph embedding method of *FlavorGraph* learns not only the food-chemical molecule relations, but also the relations between food ingredients in a large number of recipes. The recipe&novelty recommendation of food pairings (right table in Table 4) shows that *FlavorGraph* representations can be used to recommend complementary and novel pairings of foods in cooking. In this case, we only retrieved non hub ingredient nodes from FlavorGraph. This was to compare it with KitcheNette model, which has no chemical information. The results of KitcheNette, which predicts the recipe co-occurrence probability of food ingredient pairs, are ranked using Siamese neural networks. *FlavorGraph* can be used to recommend ingredients that are highly likely to be used together in recipes. For example, whole_wheat_hamburger_bun(0), bacon_piece(0), ranch_salad_dressing(0), and so on are highly ranked food ingredients that go well with tomato. In summary, *FlavorGraph* can be used to recommend pairings of food ingredients that are similar in chemical structure and most likely to be used together in recipes.

**Table 4:** 2-way food pairings based on the similarity search of food representations generated by *FlavorGraph*.

Chemical recommendation of food pairings				Recipe&novelty recommendation of food pairings			
FlavorGraph (All nodes)	FlavorDB	FlavorGraph (All nodes)	FlavorDB	FlavorGraph (Non hub nodes only)	KicheNette	FlavorGraph (Non hub nodes only)	KicheNette
Graph embedding	# of shared molecules	Graph embedding	# of shared molecules	Graph embedding	Siamese neural network prediction	Graph embedding	Siamese neural network prediction
**Tomato**		**Onion**		**Tomato**		**Onion**	
lettuce(119)	tea(186)	garlic(120)	cocoa(120)	whole_wheat_hamburger_bun(0)	lettuce(119)	frozen_lima_bean(0)	bay_leaf(0)
cucumber(115)	potato(161)	potato(119)	garlic(120)	miracle_whip_light(0)	avocado(122)	sweet_green_pepper(0)	celery(103)
avocado(122)	mango(160)	celery(103)	peanut(120)	sweet_green_pepper(0)	cucumber(115)	smoked_cheddar_cheese(0)	ground_beef(0)
onion(118)	guava(158)	chive(118)	potato(119)	serrano_chili_pepper(0)	bean_dip(0)	lean_ground_beef(0)	potato(119)
potato(161)	apple(158)	tomato(118)	tomato(118)	yellow_sweet_pepper(0)	eggplant(109)	beef_stew_meat(0)	carrot(106)
cheese(59)	grape(152)	pepper(96)	chive(118)	spam(0)	turmeric_powder(0)	canned_tomato(0)	tomato_paste(0)
jalapeno(0)	soybean(151)	cabbage(112)	soybean(117)	ears_of_corn(0)	garam_masala(0)	serrano_ham(0)	beef_broth(0)
cumin(105)	strawberry(149)	carrot(106)	green_beans(115)	bacon_piece(0)	red_chili_powder(0)	stewed_tomato(0)	beef_stock(0)
cheddar_cheese(67)	cocoa(149)	mushroom(111)	tea(115)	ranch_salad_dressing(0)	tostada(2)	green_pepper(0)	green_pepper(0)
red_bell_pepper(0)	mushroom(149)	green_bean(115)	leek(114)	taco_shell(0)	taco_shell(0)	anaheim_chilies(0)	stewing_beef(0)
**Pepper**		**Cinnamon**		**Pepper**		**Cinnamon**	
green_bell_pepper(3)	ginger(160)	nutmeg(138)	pepper(144)	round_steak(0)	oregano(149)	apple_pie_filling(0)	allspice(124)
sweet_basil(5)	laurel(159)	vanilla(104)	ginger(141)	garlic_salt(0)	ground_beef(0)	real_vanilla_extract(0)	clove(129)
red_bell_pepper(3)	rosemary(154)	allspice(124)	laurel(141)	frozen_corn_kernel(0)	potato(114)	canned_pumpkin_puree(0)	raisin(6)
cumin(136)	basil(151)	clove(129)	basil(139)	corn_flake_crumb(0)	thyme(130)	light_margarine(0)	baking_soda(0)
orange_bell_pepper(3)	spearmint(149)	walnut(102)	rosemary(138)	sweet_onion(0)	elbow_macaroni(0)	pumpkin_puree(0)	apple(113)
oregano(149)	oregano(149)	mace(18)	nutmeg(138)	browning_sauce(0)	basil(151)	canned_pumpkin(0)	nutmeg(138)
yellow_bell_pepper(3)	nutmeg(149)	raisin(6)	oregano(134)	dark_sesame_oil(0)	celery(146)	sour_milk(0)	applesauce(0)
basil(151)	orange(148)	pecan(105)	cassia(133)	soft_breadcrumb(0)	onion(96)	solid_pack_pumpkin(0)	brown_sugar(0)
parsley(140)	celery(146)	shortening(2)	tea(131)	frozen_hash_brown_potato(0)	hamburger(4)	mashed_sweet_potato(0)	pumpkin_puree(0)
carrot(141)	dill(145)	sugar(4)	celery(130)	crushed_tomato(0)	marjoram(145)	quick_oat(0)	canned_pumpkin(0)

The number of chemical compounds shared by two food ingredients is in parentheses.

*Recommending pairings of various foods* In Table 5, we demonstrated that our learned food representation vectors can also be used to provide pairing recommendations based on the combination of multiple learned food representation vectors. First, we made pairing recommendations for a single food ingredient (e.g. ice_cream, white_wine). We showed that *FlavorGraph* can recommend complementary ingredients (e.g., caramel_sauce, cookie, cake, candy for ice_cream and sole_fillet, tomato, chicken for white_wine) that would generally go well with given food ingredients. Also, we added two or more ingredient representation vectors, and used them as a pairing query vector. For example, strawberry and chocolate were added to ice_cream, and shrimp was added to white_wine. We simply summed the two vectors, and performed the similarity search on the summed result. For the ranking of the summed vector of [ice_cream+strawberry], strawberry-related desserts (e.g. strawberry_jello_o, strawberry_gelatin), or other desserts (e.g., brownie, whipped_topping, hot_fudge) that pair well with ice cream and strawberries were recommended. For the ranking of the summed vector of [ice_cream+chocolate], the recommendation results include additional desserts that go well together. White_wine is generally known to go well with seafood, vegetables, and chicken. For the ranking of summed vector of [white_wine+shrimp], different types of seafood that go well with the combination of white_wine and shrimp are recommend.

**Table 5:** Pairing recommendations for various food ingredients using simple vector arithmetic.

Pairing query	Result description	Top 20 pairing recommendations
ice_cream	general pairing for ice_cream	ice_cream, caramel_sauce, chocolate_fudge_topping, cookie, cake, candy, chocolate_syrup, chocolate_frosting, candy_sprinkle, oreo_cookie, jello_gelatin, licorice, caramel_ice_cream_topping, baileys_irish_cream, dream_whip, chocolate_wafer_cookie, heath_candy_bar, food_coloring, angel_flake_coconut, amaretto
ice_cream + strawberry = ?	additional dessert pairing for ice_cream and strawberry	brownie, fresh_rhubarb, ice_cream, strawberry_jell_o_gelatin_dessert, non_dairy_whipped_topping, strawberry_gelatin, hot_fudge, cake, strawberry_preserve, gelatin, shortbread_cookie, chocolate_hazelnut_spread, digestive_biscuit, jello_gelatin, caramel_sauce, cookie, butter_flavored_cooking_spray, amaretto, vanilla_bean_paste, pie_crust
ice_cream + chocolate = ?	additional dessert pairing for ice_cream and chocolate	ice_cream, instant_malted_milk_powder, baileys_irish_cream, espresso_powder, cookie, chocolate_liqueur, bittersweet_chocolate, creme_de_menthe, mint_extract, caramel_sauce, chocolate_sprinkle, chocolate_frosting, chocolate_syrup, chocolate_chip_cookie, licorice, chocolate_fudge_topping, green_food_coloring, graham_cracker_crumb_crust, cake, food_coloring
white_wine	general pairing for white_wine	white_wine, sole_fillet, linguine, san_marzano_tomato, chicken_cutlet, red_wine, center_cut_pork_chop, tagliatelle_pasta_noodle, chicken_breast_tender, cream_sherry, capellini, salt_cod_fish, angel_hair_pasta, lemon_slice, lemons,_zest_of, sea_bass, condensed_golden_mushroom_soup, flounder_fillet, steak, baby_portabella_mushroom
white_wine + shrimp = ?	additional seafood pairing for white_wine	white_wine, sole_fillet, linguine, cod_fish_fillet, angel_hair_pasta, lobster_tail, capellini, clam_juice, scallop, cod, flounder_fillet, bottled_clam_juice, octopus, lobster_meat, red_snapper, littleneck_clam, sea_bass, jumbo_shrimp, tartar_sauce, halibut

### Case study 2: Can we also predict compound-food relations?

**Figure 5:**
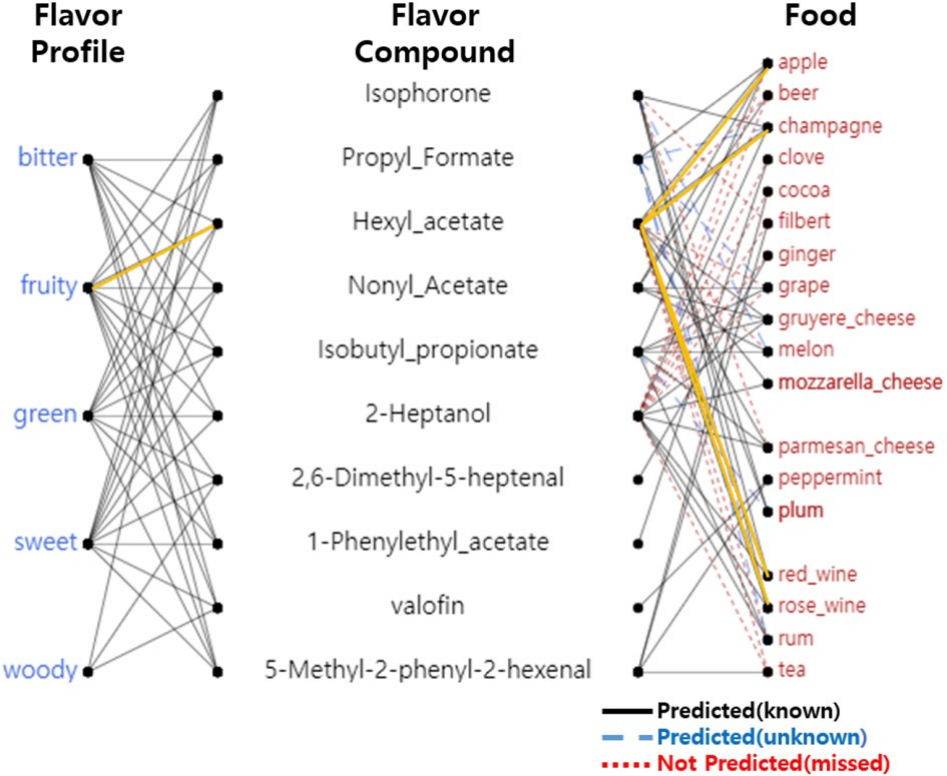
<Flavor profile-Flavor compound-Food> relation network.

*Predicting compound-food relations* When our modified version of metapath2vec is trained on all the nodes of *FlavorGraph*, the method trains not only the relations between foods but also the relations between foods and chemical compounds. Therefore, we believe that it is possible to predict pre-existing compound-food relations and undiscovered ones through a similarity search of our learned food representation vectors. We demonstrated a toy example task to predict relationship between compounds and foods. Figure 5) shows the prediction results of <Flavor profile-Flavor compound-Food> network. To build this relation network, we first picked the 5 most frequently appeared flavor profiles (out of 582) in FlavorDB^3^. We then randomly sampled ten of each corresponding random flavor compounds (out of 1561) upon the picked flavor profiles. Lastly, we randomly sampled 20 food ingredients (out of 6653) for each of the flavor compounds.

The relation edges (grey lines) on <Flavor profile-Flavor compound> were defined based on already known connections found in FlavorDB. The relation edges (grey, blue, red lines) on <Flavor compound-Food> were defined based on whether the similarity search score of two nodes exceeds a certain threshold. This threshold is further discussed in M2 of Supplementary Information. In Table 6, we illustrated the evaluation results on similarity search between food nodes and flavor compound nodes. The grey (straight) lines are the relation edges that were correctly predicted to be connected. The red lines (dotted) are the relations edges that were predicted to be not connected but actually are connected according to the dataset. The blue (dashed) lines are the relations that were predicted to be connected but are unknown up to recent discoveries. In summary, not only did the similarity search predict the existing relations with a fairly high accuracy, but also discover six new <Flavor compound-Food> relations that have not been found before. These newly discovered relations can be used to discover new compound-food relations that have not been previously identified due to cost limitations or limited access to natural food sources.

**Table 6:** Evaluation on <Flavor compound-Food> relation prediction.

# of relation	53	Accuracy	0.895
# of prediction	44	Precision	0.864
Predicted (known)	38	Recall	0.717
Predicted (unknown)	6	F1	0.784
Not predicted (missed)	15	MCC	0.720

*What are the flavor profiles related to food?* As shown in Fig. 5, we added flavor profiles related to existing <Flavor compound-Food> relations. Here, flavors such as fruity, bitter, fatty, floral, and so on are flavor compounds. Knowing and finding Food-Flavor compound-Flavor profile> relations can help in better understanding the chemical effects of food ingredients on to other food ingredients. For example, we can find foods with fruity flavor compounds based on the predicted relations between *Fruity* (flavor profile) and *Hexyl_acetate* (flavor compound). According to reference search^25^, *Hexyl_acetate* is known to be used as a flavoring because of its fruity odor, and it is naturally present in many fruits (such as apples and plums) as well as alcoholic beverages. We found that small quantities of *Hexyl_acetate* are found in a wide variety of fruits and foods including rose_wine, red_wine, champagne, apple, and parmesan_cheese. While the example results we provided here are toy examples of <Flavor profile-Food> relations, we expect to provide hints for future food researches such as flavor-specific searches.

## Conclusion

We collected food recipes and chemical information of food to build a large-scale food-compound network graph called *FlavorGraph*. We used the food-specific metapath graph embedding method with an chemical structure learning layer to generate elaborate food representation vectors for *FlavorGraph*. Then we demonstrated that the food representation vectors can be used for making food pairing recommendations and predicting new food-compound relations. However, our work has some limitations; first, more food-related information is needed to better understand food. This work combines only recipe co-occurrence information and chemical information. Second, since the metapath-based graph embedding method uses unsupervised learning, it is difficult to evaluate the food repre sentations. Last, the food pairing recommendations and food-compound relation predictions made in this study have not yet been scientifically evaluated because there is no single correct answer set that can be used for verifying such results. Nevertheless, we believe that *FlavorGraph* can be employed for better understanding the cooking and medicinal uses of food. Also, the deep learning strategies outlined in this paper can serve as the cornerstone for food pairing and food-relation prediction tasks.

## Methods

### Building *FlavorGraph*

We combined various datasets used in several food-related studies for building *FlavorGraph* which is a very large graph containing food–food and food-chemical compound relations (Fig. 1). *FlavorGraph* is comprised of three different types of nodes (e.g., food ingredients, flavor compounds, and drug compounds), and three different types of edges (e.g., food ingredient-food ingredient relations, food ingredient-flavor compound relations, and food ingredient-drug compound relations). Further details of our constructed graph are provided in Table 7.

**Table 7:** FlavorGraph—nodes and edges.

Data source		Nodes 8298	Edges 147,179
Im2recipe	I Ingredient 6653	H Chemical-hub Ingredient 416	I–I Ingredient–Ingredient 111,355
		N Non-hub Ingredient 6237	
FlavorDB	C Compound 1645	F Flavor compound 1561	H–F Chemical-hub ngredient-Flavor Compound 35,440
HyperFoods		D Drug compound 84	H–D Chemical-hub Ingredient–drug compound 386

#### Food-food relations

We extracted the ingredient nodes (I) and ingredient–ingredient relations (I–I) from Recipe1M^16,22^ which is a large-scale dataset of human-written cooking recipes. 16,857 unique candidate ingredients were selected from the recipes and co-occurring probabilities of all ingredient pairing combinations were calculated. The pairing scores were calculated based on the co-occurring probabilities. The higher the co-occurring probability of two ingredients, the higher their pairing score. This scoring approach was first introduced in the work by Teng et al.^5^ where Pointwise Mutual Information (PMI) (Eq.(1)) was used to create a simple ingredient network. Similarly, a normalized version of PMI (NPMI)^26^ was utilized for training the pairing score prediction model KitcheNette^13^.

1 $$\begin{aligned} {\text {pmi}}(x;y) = \log \frac{p(x,y)}{p(x)p(y)} ,\quad \quad p(x,y) = \frac{\# \hbox { of recipes where x and y occur together}}{\# \hbox { of recipes}} \end{aligned}$$

As done in the work by Park^13^, we used NPMI^26^ scores to construct edges between every two ingredient nodes in FlavorGraph. Among all the possible candidate ingredient pairs from the list of extracted ingredients, we included only highly complementary candidates in our network. Candidate ingredient pairs satisfying one of the following conditions were selected as network edges: (1) Each ingredient appears more than 20 times in Recipe1M and both of them appear more than 5 times in the same recipe. (2) The calculated NPMI score is at least 0.25. Ingredient pairs whose NPMI score is less than 0.25 but ranked in the top 20 below the 0.25 threshold were also included as edges in FlavorGraph. A total of 7199 ingredient nodes and 164,531 ingredient-ingredient edges were included in FlavorGraph.

#### Food-chemical compound relations

For flavor compounds (F) and ingredient–flavor compound relations (I-F), we used FlavorDB^3^ to create chemical edges in FlavorGraph. FlavorDB collates information from several different food-related databases (e.g., FooDB^27^, Flavornet^28^) which contain a list of flavor molecules from natural food ingredients. It also gathers chemical information on flavor molecules such as bitter substances (BitterDB^29^), sweet substances (SuperSweet^30^), scents (SuperScent^31^), nutritional factors (NutriChem^32^), polyphenols (Phenol-Explorer^33^), and so on. FlavorDB contains 2254 flavor compounds found in 936 natural food ingredients. 400 of the 2254 flavor compounds were selected as they are included in the ingredient nodes built from Recipe1M. We found that the 400 ingredients (e.g., chicken, rice, banana) are popularly used in cooking while the rest of them (e.g., hyacinth_bean, mammee_apple, drumstick_leaf) are rarely used. Based on the 400 selected ingredient nodes that have flavor compound information, there is a total of 1561 flavor compound nodes and 164,531 ingredient-flavor compound edges.

For drug compounds (D) and ingredient–drug compound (I–D) relations, we used HyperFoods^23^ to create chemical edges in graph. HyperFoods exploits machine learning to map cancer-beating drug compounds to natural food ingredients. HyperFoods is trained on drug-gene relations to infer food-gene relations by means as suggesting remedial foods for cancer treatment/prevention. 206 natural food ingredients are provided in a dataset used in HyperFoods but we used only 104 of them for constructing FlavorGraph. Based on the 104 ingredient nodes that have drug compound information, there are 84 drug compound nodes and 386 ingredient-drug compound edges.

### Graph node embedding in FlavorGraph

We employed metapath2vec^21^ which can learn representations of ingredient, flavor compound, and drug compound nodes, and the relations between the nodes. In metapath2vec, the nodes of FlavorGraph are trained to learn relations between ingredients in food recipes and the chemical information of ingredients.

#### Generating metapaths from FlavorGraph

The scientific approach to food pairing focuses on the number of shared compounds as explained in Ahn’s work^1^; however, this approach suffers from the limited availability of chemical information on food ingredients (H—chemical-hub ingredients in *FlavorGraph*). To overcome this problem, we aim to design to learn chemical information (F, D) and even information on non-hub ingredient nodes (N). Food-specific metapaths are generated from FlavorGraph so that the chemical compound nodes (C) can pass information to non-hub ingredient nodes (N) through chemical-hub ingredient nodes (H). To do so, we first set up metapaths (e.g. C–H–N–H’–C’) starting from compounds (C) and ending at compound nodes (C) so that the ingredient nodes (H, N, H) in the path can share the same context. For the nodes of the same type shared in a path are set to have different elements meaning that C and C’ are different compounds. In the same way, we added metapaths (e.g. N–H–C–H’–N’) starting at non-hub ingredients (N) and ending at N. These metapaths were added so that every non-hub ingredient node (N) is trained at least once and learns chemical information. Last, to reflect all nodes in a balanced way, node2vec^20^ based on completely random walks was added. The number of walks of each starting node was set to 100 and the maximum length of each metapath was set to 50. A total of 1,114,285 metapaths whose average length is 46.36 were used for final learning.

#### metapath2vec

As done in the work of Dong^21^, we applied the skip-gram model to the generated metapaths in order to generate node representations. For each metapath, we maximized the likelihood of each node *u* to its heterogeneous context (C–H–N–H’–C’ or N–H–C–H’–N’) *W(u)* where *W(u)* denotes the other nodes within a fixed window size of the metapath:

2 $$\begin{aligned} \arg \max _{\theta } \sum _{u\in P} \sum _{c_u\in W(u)}\log p(c_u|u;\theta ) \end{aligned}$$

where $\log {p(c_u|u;\theta )}$ is defined as a softmax function $\frac{e^{X_{c_u}\cdot {X_u}}}{\sum _{v \in V}{e^{X_v\cdot X_u}}}$, where Xi is the *i*th embedding vector in X.

We also employed negative sampling where a set of M words is sampled from FlavorGraph. Therefore, the above equation is updated as

3 $$\begin{aligned} \log \sigma (X_{c_u}\cdot X_{u}) + \sum _{m=1}^{M}{\mathbb {E}}_{u^{m}\sim Q(u)}[\log \sigma (-X_{u^m}\cdot X_{c_u})] \end{aligned}$$

where $\sigma {(x)}$ is $\frac{1}{1+e^{(-x)}}$ and *Q* is a distribution from where $u^m$ is drawn *M* times. The graph embedding method was used to learn various contexts from different types of nodes in generated food-specific metapaths of FlavorGraph.

#### metapath2vec with chemical structure prediction (CSP) layer

**Figure 6:**
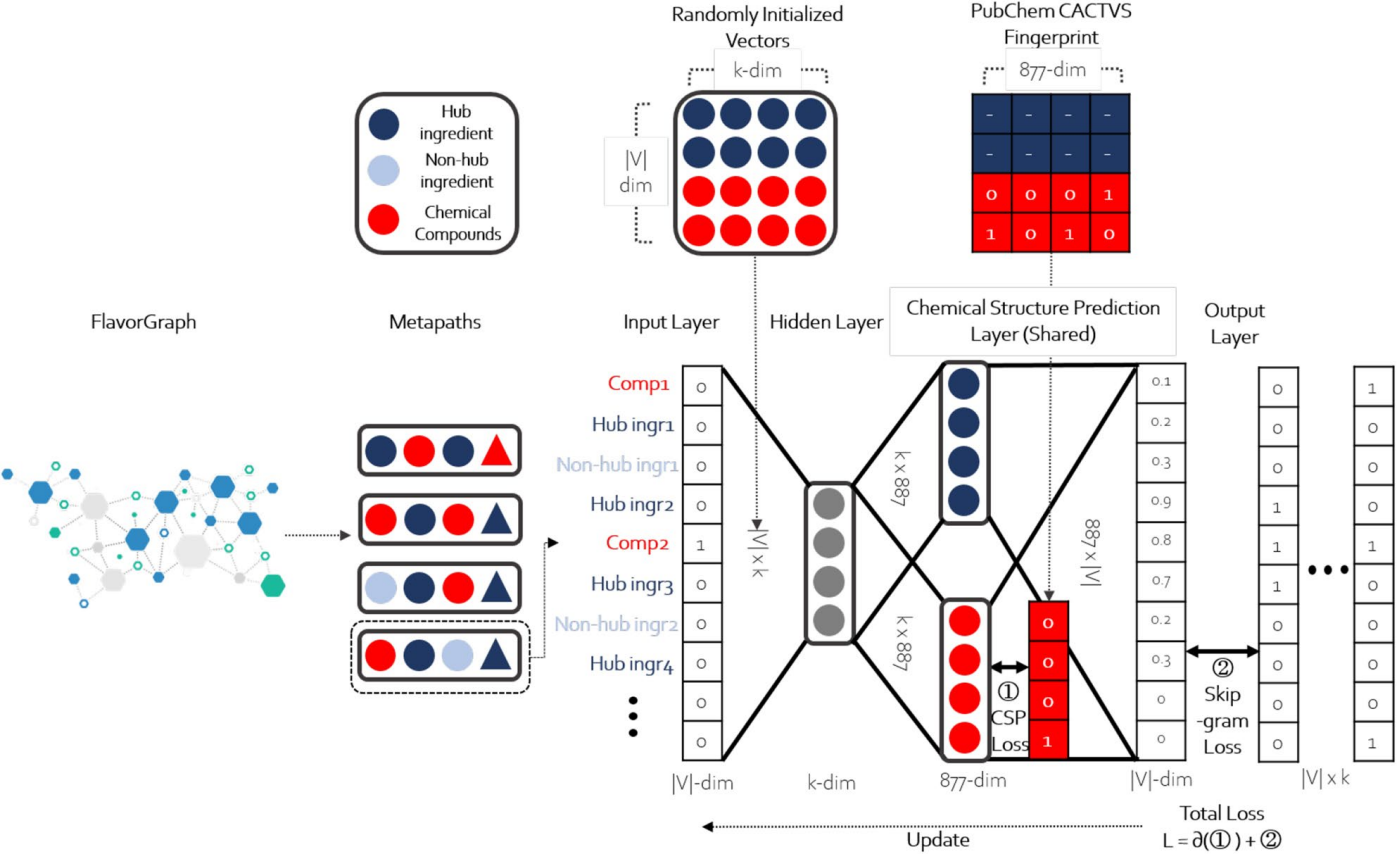
Graph Embedding with metapath2vec+CSP on FlavorGraph.

As shown in Fig. 6, we implemented upgrade version of metapath2vec with chemical structure prediction (CSP) layer. As the food-specific metapaths in FlavorGraph have not only non-hub ingredient nodes (N), chemical-hub ingredient nodes (H) but also flavor compound nodes (F) and drug compound nodes (D), we included chemical knowledge in our flavor representations. However, even though the flavor and drug compound nodes have chemical structure information represented as CACTVS fingerprints. More information is available from PubChem Fingerprints), their valuable chemical information is not taken into account in learning. CACTVS fingerprints are expressed as 881-dimensional binary vectors where each element of the binary vectors indicates whether a particular molecular substructure exists in a certain chemical compound. Each bit of a fingerprint represents the presence or absence of one of the 881 chemical substructures. A chemical structure prediction (CSP) layer which is designed to use the more detailed information on chemical compounds was added to the original skip-gram model. We expect the skip-gram model to learn the available chemical structure information on flavor&drug compounds and use compound-ingredient relations to generate more significant node representations.

With the encoded vectors from the CSP layer, the graph embedding method was trained to accurately predict the underlying chemical structure of flavor and drug compounds exclusively. The loss function for the CSP layer is the binary cross entropy between the encoded vectors from the CSP layer and the binary fingerprint vectors corresponding to chemical compounds. The CSP loss function is added to the graph embedding method as follows:

4 $$\begin{aligned} \log \sigma (X_{c_u}\cdot X_{u}) + \sum _{m=1}^{M}{\mathbb {E}}_{u^{m}\sim Q(u)}[\log \sigma (-X_{u^m}\cdot X_{c_u})] + \lambda {\frac{1}{D}\sum _{d=1}^{D}({\mathbf{y}} _d\log {f_d(u)} + (1-{\mathbf{y}} _d)\log ({1 - f_d(u))})} \end{aligned}$$

where $f_d$ is the chemical encoder function for the *d*th dimension of the *D*-dimensional encoded vector from the CSP layer, ${\mathbf{y}} _d$ is the binary label for *d*th chemical substructure in the actual CACTVS vector and $\lambda$ is the weighting factor ranging from 0.0 to 1.0. Note that *D* is 881 which is the actual number of chemical substructures in our CACTVS/PubChem fingerprints. As the gradient backpropagates through both the CSP layer and the skip-gram model, we expect our updated embedding ingredient vectors to contain chemical information. However, not all ingredient vectors have such information due to the limited coverage of available databases. While the skip-gram model will make predictions for all ingredients, only those with 881-dimensional binary vectors as labels were included in the additional cross entropy loss. Therefore, the CSP task is trained in a semi-supervised learning setting. In sum, the skip-gram model is used for learning contextual information in pairing paths, and the CSP layer is used for learning chemical structure information.

## Supplementary Information

Supplementary Information.

## Data Availability

We set up a public repository for running our model and obtaining results. Data and trained food representations are available at https://github.com/lamypark/FlavorGraph. The repository contains graph data with node information on food ingredients and chemical compounds and edge information on their relations. Our graph embedding model is implemented and tested on Python 3.5.2, PyTorch 1.0.0, and CUDA 9.0. The hardware specifications of our server are as follows: Intel Xeon(R) E5-2630 v4@2.2GHz CPU with 128GB memory, GTX Titan X GPU with 12GB memory. The time for training our graph embedding model per epoch is about 180 seconds.
