# Supplementary material for: FlavorGraph: a large-scale food-chemical graph for generating food representations and recommending food pairings
Source: Sci Rep. 2021 Jan 13;11:931. doi: 10.1038/s41598-020-79422-8 (PMC7806805; doi:10.1038/s41598-020-79422-8)
Supplement: Supplementary file 1 — Supplementary Information. [file 41598_2020_79422_MOESM1_ESM.pdf]

Supplementary Information for FlavorGraph: A large-scale food-chemical graph for generating food representations and recommending food pairings

Donghyeon Park[a], Keonwoo Kim[a], Seoyoon Kim[a], Michael Spranger[b], and Jaewoo Kang[a]

[a]145 Anam-ro, Seongbuk-gu, Seoul, 02841, South Korea
[b]1 Chome-7-1 Konan, Minato City, Tokyo 108-007, Japan

Correspondence: Jaewoo Kang (kangj@korea.ac.kr)

This PDF file includes:

Supplementary methods M1 to M2

Supplementary methods (M1): Hyper-parameter Sensitivity for Generating Metapaths and Building Graph Embedding Model

0.1 Hyper-parameter for generating metapaths

In Fig. 1, we report few different hyper-parameters when generating metapaths to train our graph embedding methods. In Fig. 1a, we tried five different (10, 50, 100, 250, 500) "# of walks per node" in *FlavorGraph*, the model achieved the highest score in NMI clustering task when it was 100. In Fig. 1b, we tried five different (10, 25, 50, 100, 200) "length of metapath" in *FlavorGraph*, the model achieved the highest score when it was 50.

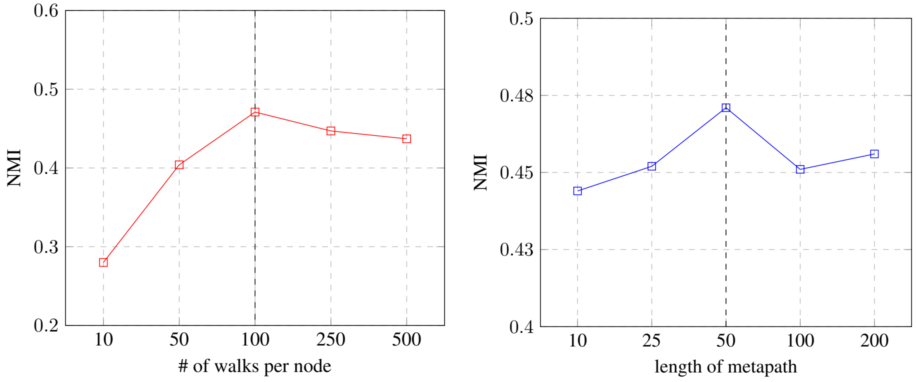

(a) # of walks per node

(b) length of metapath

Figure 1. Compound-Food relation prediction according to similarity threshold.

0.2 Hyper-parameters for graph embedding model.

For fair comparison, we set same hyper-parameters on baseline models (node2vec, metapath2vec, metapath2vec+CSP) based on skip-gram. The dimension of embedded vectors was set to 300, the learning rate was set to 2.50E-02, the window size of set to 3, and the minimum count of learning was sset to 3.

Supplementary methods (M2): Threshold on Predicting Compound-Food Relation.

The threshold for the similarity search on compound-food relations was chosen by the following metrics, as illustrated in Fig. 2. For flavor compound-food relations (Fig. 2a, the F1 score and the Matthews correlation coefficient (MCC) score of the similarity search experiment were highest at the threshold of 0.3. For drug compound-food relations (Fig. 2b, the F1 score and the Matthews correlation coefficient (MCC) score of the similarity search were highest at the threshold of 0.4. Thus, we selected thresholds of 0.3 and 0.4 for our similarity search and prediction of compound-food relations.

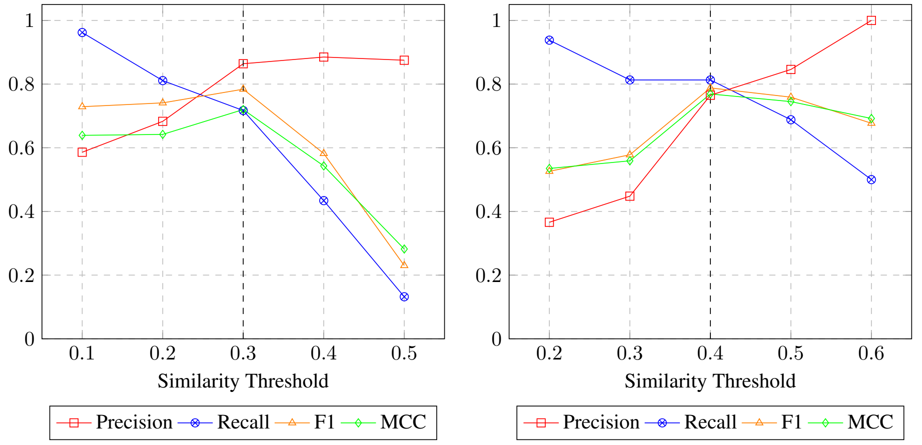

(a) Flavor compound-food link prediction

(b) Drug compound-food link prediction

Figure 2. Compound-Food relation prediction according to similarity threshold.
